# Pharmacist–Physician Collaboration to Improve the Accuracy of Medication Information in Electronic Medical Discharge Summaries: Effectiveness and Sustainability

**DOI:** 10.3390/pharmacy8010002

**Published:** 2019-12-30

**Authors:** Rohan A. Elliott, Yixin Tan, Vincent Chan, Belinda Richardson, Francine Tanner, Michael I. Dorevitch

**Affiliations:** 1 Pharmacy Department, Austin Health, Heidelberg, VIC 3084, Australia; yixin.tan@waitematadhb.govt.nz (Y.T.); vincent.chan@rmit.edu.au (V.C.); belinda.richardson@austin.org.au (B.R.); francine.tanner@austin.org.au (F.T.); 2 Centre for Medicine Use and Safety, Monash University, Parkville, VIC 3052, Australia; 3 Pharmacy Department, Waitemata District Health Board, Auckland 0620, New Zealand; 4 School of Health and Biomedical Sciences, RMIT University, Bundoora, VIC 3083, Australia; 5 Aged Care Services, Austin Health, Heidelberg, VIC 3084, Australia; michael.dorevitch@austin.org.au

**Keywords:** care transition, patient transfer, patient discharge summaries, pharmacists

## Abstract

Inaccurate or missing medication information in medical discharge summaries is a widespread and intractable problem. This study evaluated the effectiveness and sustainability of an intervention in which ward-based hospital pharmacists reviewed, contributed and verified medication information in electronic discharge summaries (EDSs) in collaboration with physicians. Retrospective audits of randomly selected EDSs were conducted on seven wards at a major public hospital before and after implementation of the intervention and repeated two years later on four wards where the intervention was incorporated into usual pharmacist care. EDSs for 265 patients (prescribed a median of nine discharge medications) were assessed across the three time points. Pharmacists verified the EDSs for 47% patients in the first post-intervention audit and 68% patients in the second post-intervention audit. Following the intervention, the proportion of patients with one or more clinically significant discharge medication list discrepancy fell from 40/93 (43%) to 14/92 (15%), *p* < 0.001. The proportion of clinically significant medication changes stated in the EDSs increased from 222/417 (53%) to 296/366 (81%), *p* < 0.001, and the proportion both stated and explained increased from 206/417 (49%) to 245/366 (67%), *p* < 0.001. Significant improvements were still evident after two years. Pharmacists spent a median of 5 (range 2–16) minutes per patient contributing to EDSs. Logistics, timing and pharmacist workload were barriers to delivering the intervention. Additional staff resources is needed to enable pharmacists to consistently deliver this effective intervention.

## 1. Introduction

Medical discharge summaries, or discharge letters, are a key communication tool for patient safety [1]. Australian national indicators for quality use of medicines in hospitals state that all discharge summaries should include “a current, accurate and comprehensive list of medicines” and “medication therapy changes and explanations for changes” [2].

It is widely recognised, in Australia and internationally, that discharge summaries prepared by hospital physicians (including medical interns, junior medical officers and senior medical staff) often include incomplete or incorrect medication information [1,3,4]. Medication lists may be inaccurate, and medication changes made in hospital are often not documented, or they may be documented but not explained [3,5,6,7,8,9,10]. Failure to accurately communicate discharge medication information increases the risk of post-discharge prescribing errors, suboptimal monitoring of new medications, and unplanned re-hospitalisation [3,11,12,13,14].

Many strategies to improve the quality of medication information in discharge summaries have been trialed, including guidelines, performance indicators, physician education and discharge summary templates with specific sections for medication information [3,8,15]. However, poor quality of discharge medication information persists [1,4,8,16]. Electronic discharge summaries (EDSs) have the potential to improve the accuracy of discharge summary data, especially if they are integrated with electronic prescribing systems, but studies comparing EDSs with handwritten discharge summaries have produced variable results [17]. Several studies evaluating the accuracy of EDSs have reported high levels of missing or inaccurate information [10,16,18]. For example, our group evaluated an EDS in which discharge medication lists were imported from the hospital’s e-prescription record, and medication changes were manually typed [10]. We found that 59% of EDS had medication list discrepancies, and only 34% of clinically significant medication changes were explained [10]. Medication list discrepancies occurred when, for example, over-the-counter medicines were not included on the discharge prescription, medication changes were made in the e-prescribing system after the EDS was prepared, or amendments were made on the paper copy of the e-prescription that was sent to the hospital pharmacy for dispensing [10]. Similarly, an Australian study by Gilbert et al. reported medication list discrepancies in 31% of EDSs and no reporting of reasons for medication changes despite there being a ‘medication changes’ table in the EDS template [16].

Another approach for improving the quality of medical discharge summaries is to have ward-based hospital pharmacists prepare or review the medication information sections. Pharmacists in Australia, and many other countries, are well placed to perform this activity as they already routinely perform medication reconciliation on discharge to ensure discharge medication orders are accurate, and to identify medication changes in order to communicate these to patients, carers and community pharmacies. There is evidence that pharmacists can prepare discharge medication information with a high degree of accuracy [10,19,20], and that pharmacist-led medication reconciliation leads to fewer medication discrepancies at discharge [21]. A number of studies have highlighted the benefits of involving pharmacists in preparing medical discharge summaries [18,22,23,24,25]. Studies by Alex et al. [18], Bergvist et al. [22] and Tong et al. [23] demonstrated that pharmacists’ input into discharge summaries led to more accurate medication lists, while studies by Ooi et al. [24] and Duedahl et al. [25] demonstrated that pharmacists’ input led to more accurate information about medication changes. In their randomised controlled trial, Tong et al. [23] reported an absolute risk reduction of 46.5% for medication list errors when pharmacists completed the medication section of patients’ discharge summaries, compared with standard physician-prepared discharge summaries. Ooi et al. [24] reported that a greater proportion of medication changes were documented in pharmacist-prepared discharge medication summaries compared with physician-prepared discharge summaries (72.8% vs. 31.5%).

It is important to provide both an accurate medication list and explanations for medication changes made in hospital. No studies to our knowledge have evaluated the impact of pharmacists on both of these outcomes, nor the sustainability of pharmacist discharge summary interventions. Contributing to medical discharge summaries adds to pharmacists’ workload, and whilst improvement may be observed in short-term intervention studies, it is important to know whether these can be sustained in a busy hospital environment and with staff turnover.

The aims of this study were to evaluate the effectiveness of an intervention in which ward-based pharmacists reviewed, contributed and verified medication information in patients’ EDSs, and determine whether improvements were sustained two years after the initial post-intervention audit.

## 2. Materials and Methods

### 2.1. Setting and Study Design

This was a pre- and post-intervention study that used retrospective medical record audits to assess the accuracy of EDSs for random samples of patients discharged from inpatient wards at a major public teaching hospital in Melbourne, Australia, at three time points: 2014, 2015 and 2017. The methods used for the audits in this study were the same as those used in a previous hospital-wide audit of EDSs at our hospital [10].

The intervention was piloted on seven wards (four aged care wards, one general medicine ward, one surgical ward and one psychiatry ward—total 188 beds) for 10 weeks from April 2015. It was integrated into routine care after the pilot study on the four aged care wards (total 104 beds) but was unable to be sustained on the other three wards due to resource constraints.

The study was originally conceived as a pre- and post-intervention study involving the seven pilot wards in 2014 and 2015. Two years later (in 2017) we repeated the audit on the four wards that continued to receive the intervention, with the help of final-year pharmacy students, to provide data on sustainability of the intervention. The study was approved by our hospital’s Human Research Ethics Committee.

### 2.2. Electronic Information and Prescribing Systems Used in the Study Hospital

Prescribing and EDS preparation occurred within an electronic medical record (Cerner Millennium). Inpatient medication administration records were electronic (paperless), while discharge prescriptions were prepared electronically then printed on paper.

### 2.3. Preparation of EDSs Prior to the Intervention

The process for producing discharge prescriptions and EDSs was as follows [10]:

1. A hospital physician (usually an intern or junior medical officer) prepared a discharge prescription, then printed and signed the paper copy. Hospital policy was that all medications intended to be taken after discharge were to be included on the prescription, regardless of whether or not they needed to be dispensed, to ensure an accurate electronic record;
2. A hospital pharmacist reviewed the paper discharge prescription and performed medication reconciliation by comparing the prescription with the patient’s inpatient medication administration record and pre-admission medication history (which had been recorded and verified by a pharmacist upon admission to hospital) to identify unintended discharge prescription discrepancies (e.g., omitted medications, unnecessary medications, dose errors, dose-form errors);
3. The pharmacist discussed discrepancies with the prescriber, and amendments to the discharge prescription were agreed:
  - For amendments that did not require a new paper prescription (e.g., cessation of medication, addition of medication that did not need to be dispensed because the patient had a supply at home, change of dosage/directions), the pharmacist and/or doctor annotated and signed the amendment on the paper prescription. The hospital doctor was expected to also make the amendment in the e-prescription record, but there was no mechanism to ensure or check that this was done;
  - For other amendments, the pharmacist requested a new prescription be printed and signed by the hospital physician;
4. Using the pharmacist-verified paper prescription, discharge medications were dispensed by the hospital’s pharmacy department. Scanned copies of the processed paper prescription were stored electronically;
5. The hospital physician, again usually a junior, prepared the EDS:
  - The electronic record of the discharge prescription was imported into the EDS by clicking on a link within the EDS;
  - Information about medication changes and reasons for changes were manually entered into the EDS.
6. The EDS was signed off by the hospital physician and automatically transmitted electronically to the patient’s primary care physician.

(Note: Steps 5 and 6 sometimes occurred immediately after step 1. If the e-prescription record was subsequently changed, the EDS was not automatically updated). 

### 2.4. Intervention

The intervention was developed and implemented in 2015. It was based on findings from the 2014 baseline audit, stakeholder consultation and literature review. The intervention involved enhancing step 5 of the process described above to include a review of the EDS discharge medication list and medication change information by ward-based hospital pharmacists in order to verify EDS accuracy and amend or add information as required. This included amending the e-prescription record to reflect the agreed medication regimen following step 3 above (with the physician to co-sign where necessary), re-importing the updated medication list to the EDS when necessary, and manually editing the EDS to add medication change information or other details related to the discharge medications. Once the pharmacist was satisfied that the information was complete and accurate, they inserted a statement that the medication list had been verified by the pharmacist and the date and time of verification.

There were six pharmacists involved in delivering clinical pharmacy services to the seven post-intervention wards in 2015. The pharmacists were asked to review and amend the EDS when they were completing their other discharge-related tasks (e.g., preparing/checking discharge medications and patient-held medication lists). Physicians on the participating wards were asked not to sign-off the EDS until the pharmacist had reviewed and verified the medication information. If the doctor signed off the EDS before the pharmacist had reviewed and updated it, and there were discrepancies or omissions, an addendum to the EDS could be created by the pharmacist, which would be automatically sent to the primary care physician. To avoid sending multiple discharge summaries unnecessarily, pharmacists were instructed not to create an addendum unless the EDS had incorrect medication information that they felt could risk patient harm.

Each pharmacist received one-on-one training from the project officer. This included how to perform the intervention, professional responsibilities associated with accessing and adding or modifying information in the EDS, and examples of when addenda should be created if the EDS was already signed off. Flow charts summarizing the process were provided. All pharmacists had been working at the study hospital for at least 2 years and had 2 to 8 years of hospital pharmacy experience.

During the initial implementation and evaluation period (April–June 2015) the project officer monitored the intervention and provided ongoing training, support and feedback for the pharmacists, in person and via regular email bulletins. Beyond 2015, when the intervention was continued on the four aged care wards, this support was no longer available. During the 2017 post-intervention audit period, four pharmacists delivered the intervention. Two had been involved in the 2015 intervention, and these pharmacists trained other pharmacists who rotated into the area.

### 2.5. Sample Selection 

Pre-intervention (baseline) and post-intervention EDS audits included patients who were discharged from the seven pilot wards between April and June 2014 and April and June 2015 respectively. A second post-intervention audit included patients discharged from the four aged care wards between April and June 2017. The audits were conducted in the second half of 2014, 2015 and 2017 respectively.

Reports of all adult patients with an inpatient stay of 24 h or more who were discharged from the participating wards during each study period were generated [10]. The number of discharges included from each ward was based on the proportion of the total discharges contributed by that ward during the period. The required sample size for each ward was entered into Microsoft Excel to generate a random number sequence to identify patients for inclusion. Additional random numbers were generated to replace patients who met exclusion criteria. Patients were excluded if they:

- were discharged to another hospital;
- died in hospital;
- did not take any medications prior to admission and were not prescribed medications on discharge;
- did not have a completed EDS in the medical record;
- had missing records that were required for the audit (e.g., pharmacist-verified “Medication History on Admission” form or pharmacist-reviewed and reconciled paper discharge prescription).

### 2.6. Data Collection

The baseline audit and the first post-intervention audit were conducted by the project officer (YT) and a co-investigator (BR) who were both hospital pharmacists. The second post-intervention audit was conducted by four final-year pharmacy students under supervision of two study investigators (YT and RE). To ensure a consistent approach to the data collection, the investigators collected data with the students for the first few patients. Thereafter the students double-checked each other’s data and the investigators performed random checks. 

To assess the accuracy of medication lists in the EDS, the EDS medication list was compared with the scanned record of the pharmacist-reviewed, reconciled and dispensed paper discharge prescription (as this was considered to be the most accurate discharge medication list, reflecting the medications the patient was intended to take after discharge). Medication discrepancies were classified as: (a) medication omission; (b) unintentional inclusion of medication; (c) strength or dose discrepancy; or (d) strength or dose omission [10].

To assess the accuracy of medication change documentation in the EDS, the pharmacist-verified “Medication History on Admission” form was compared to the scanned record of the pharmacist-verified paper discharge prescription to identify changes to patients’ pre-admission medications that had were in hospital. A medication change was defined as commencement of a new medication, cessation of a pre-admission medication or dose-modification of a pre-admission medication. The EDS was reviewed to determine whether changes had been accurately stated and whether the reason for the change was explained. If the reason for a change was not explicitly stated but was able to be easily deduced from the clinical synopsis or discharge diagnosis (e.g., commencement of a bisphosphonate for a patient who presented with a low trauma bone fracture), it was considered to have been explained [10]. 

### 2.7. Clinical Significance of Medication Changes and EDS Discrepancies 

The process by which clinically significant changes and discrepancies were determined has been described previously [10]. Briefly, it involved a review of medication changes and medication list discrepancies in a pre-intervention audit by one pharmacist investigator (YT) to identify those for which failure to accurately communicate in the EDS would be associated with low risk of an adverse outcome. These were discussed with two other investigators, one senior geriatrician (MD) and one senior pharmacist (RE), to reach expert consensus. Examples of medications for which the risk was deemed low if not accurately communicated in the EDS included aperients, lubricant eye drops and simple analgesics (excluding opioids) when prescribed ‘if required’, and non-essential supplements (e.g., multivitamins) and topical products (e.g., emollients). All other medication discrepancies and changes were classified as potentially clinically significant. This agreed classification was applied across all three audits.

### 2.8. Time Required and Barriers to Delivering the Intervention

To estimate the additional pharmacist time required to deliver the intervention, participating pharmacists were asked to document how long it took them to conduct discharge medication reconciliation activities over a 5 day period pre- and post-intervention in 2015, using a pre-piloted data collection form. Pharmacists were also asked to note barriers they encountered that prevented them from delivering the intervention. Reasons/barriers explaining why the intervention was not delivered for some patients were collated and grouped thematically by one investigator (RE), then reviewed by the other investigators.

### 2.9. Primary Outcome Measures

- Proportion of EDSs with one or more clinically significant medication list discrepancies;
- Proportion of clinically significant medication changes that were stated in the EDS;
- Proportion of clinically significant medication changes that were both stated and explained in the EDS.

### 2.10. Secondary Outcome Measures

- Number of EDS medication list discrepancies per patient;
- Types of medication list discrepancies;
- Proportion of EDSs with evidence of pharmacist verification;
- Time required by pharmacists to deliver the intervention.

### 2.11. Sample Size and Statistical Analysis

We sought to include around 100 patients in the 2014 and 2015 pre- and post-intervention audits (approximately 10% of patients discharged from the study wards). The minimum sample size required was 39 in each group, based on an estimated reduction in the proportion of EDSs with one or more clinically significant medication discrepancies from 50% to 20% (power 80%, level of significance 0.05, two sided).

Statistical analyses were conducted using GraphPad^®^ Software (GraphPad Software Inc., San Diego, CA, USA). Differences in proportions were calculated using Chi-squared tests, while Mann–Whitney U or Kruskal–Wallis tests were used to compare distributions of non-normally distributed data as appropriate.

## 3. Results

### 3.1. All Pilot Intervention Wards: Baseline (2014), Post-Intervention (2015)

#### 3.1.1. Study Sample

In the 2014 baseline and 2015 post-intervention audits, 115 and 120 patients, respectively, were reviewed. Of these, 22 and 24 patients met exclusion criteria (Table 1). A final sample of 93 and 96 patients respectively was included. Patient demographics were similar between the two groups (Table 2).

#### 3.1.2. Accuracy of Medication Lists in EDSs

**Figure 1:**
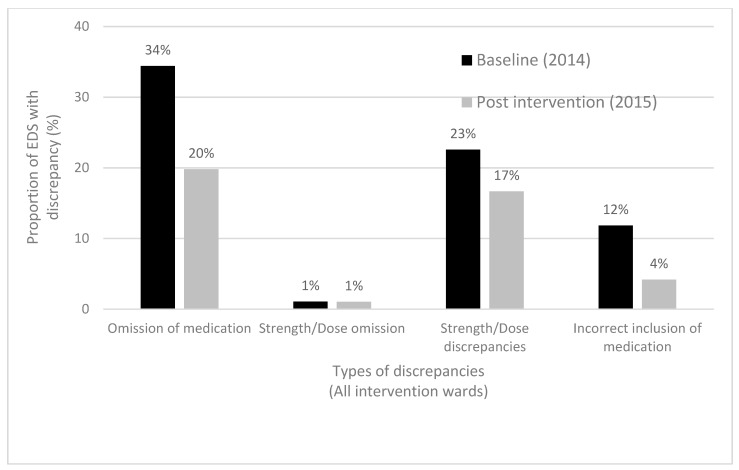
Medication list discrepancies, all pilot intervention wards.

Ninety-three and 92 EDSs in the baseline and post-intervention groups, respectively, contained a discharge medication list. In the baseline group (pre-intervention), 40/93 (43%) EDSs had one or more clinically significant medication list discrepancies, compared to 14/92 (15%) in the post-intervention group (*p* < 0.001) (Table 3). The types of discrepancies are summarized in Figure 1.

#### 3.1.3. Communication of Medication Changes in the EDS

A total of 456 and 409 medication changes were made to patients’ pre-admission medications in the baseline and post-intervention groups respectively—of which, 417 (91%) and 366 (89%) were deemed clinically significant. There were significant improvements in reporting of medication changes in the EDS following implementation of the pharmacist intervention in 2015 (Table 3).

### 3.2. Aged Care Wards: Baseline (2014), Post-Intervention (2015), Post-Intervention (2017)

#### 3.2.1. Study Sample

From the four aged care wards, in the 2014 baseline, 2015 post-intervention and 2017 post-intervention audits, a total of 46, 49 and 96 patients were reviewed respectively. Of these, 5, 7 and 20 met the exclusion criteria (Table 1), leaving a final sample size of 41, 42 and 76 respectively. Patient demographics were similar between the groups, except the number of medication changes was higher in 2017, which coincided with a longer length of stay (Table 4).

#### 3.2.2. Accuracy of Medication Lists in EDSs

All patients had a discharge medication list in their EDSs. In the 2014 baseline group, 18/41 (44%) had one or more clinically significant EDS medication list discrepancy, compared to 5/42 (12%) and 18/76 (24%) respectively in the 2015 and 2017 post-intervention groups (both *p* < 0.05 vs. baseline) (Table 5). The difference between the two post-intervention audits, in 2015 and 2017, was not statistically significant (*p* = 0.12).

#### 3.2.3. Communication of Medication Changes in the EDS

There was a total of 238, 239 and 670 medication changes in the 2014, 2015 and 2017 groups respectively—of which, 219 (92%), 212 (89%) and 612 (91%) were deemed clinically significant. There were significant improvements in reporting and explanation of changes in the EDS following implementation of the pharmacist intervention in 2015 (Table 5). Between 2015 and 2017, there was a small decline in the proportion of clinically significant changes stated in the EDS (from 87% to 76%, *p* < 0.01), but the proportion both stated and explained remained steady (Table 5).

### 3.3. Pharmacist Verification of EDS Medication Lists

The proportion of patients whose EDS medication list was verified by a pharmacist was 47% during the 2015 pilot (Table 3). It was higher on the aged care wards and did not change between the first post-intervention audit in 2015 and the repeat audit in 2017 (Table 5). 

### 3.4. Time to Deliver the Intervention

Pharmacists reported spending a median of 5.0 additional minutes per patient delivering the intervention (range 2.0–16.0 min).

### 3.5. Barriers to Delivery

The most common reasons cited by pharmacists for not delivering the intervention were that, at the time of the pharmacist’s attempt to access it, the EDS had not yet been created or the EDS was being prepared or edited (and hence was locked) by the hospital physician. Lack of time was also a barrier, especially when there was a large number of discharges on the same day, as the pharmacists were delivering the intervention in addition to their usual duties. This was especially the case for acute wards, where patient turnover was greatest and there were as many as 10 discharges on some days.

## 4. Discussion

This study demonstrated the positive impacts of pharmacist–physician collaboration in the preparation of medical discharge summaries. Significant improvements were observed in both medication list accuracy and medication change information when ward-based pharmacists reviewed, contributed and verified medication information in the EDS. Importantly, in the four wards where the intervention was incorporated into usual care, benefits were sustained two years later. This finding is important because, in contrast to the first post-intervention audit where there was a project officer supporting the intervention, the benefits observed two years later were a reflection of usual ward pharmacist practice without additional support. Furthermore, the pharmacists at that time did not know the EDS would be audited, which adds strength to the findings because there was no potential bias from a Hawthorn effect.

Unlike most previous studies that only assessed the accuracy of discharge medication lists [18,22,23], this study demonstrated additional benefits in terms of an improvement in communication of medication changes that were made in hospital. Communication of medication changes is critical at times of transition of care. A common complaint from primary care physicians is that medication changes are often not stated or explained in discharge summaries [26]. Failure to mention changes makes it difficult for the primary care physician to know whether they were intentional or accidental, and failure to explain the reasons for medication changes makes it difficult for them to know whether to re-start a ceased medication, whether to continue a new medication, whether to titrate the dose, what the treatment goal is and what outcomes to monitor. This is particularly relevant in cases where the reason(s) may not be obvious [24]. 

Documenting medication changes and their reasons is a manual process, and therefore can be time consuming, especially when a patient has been in hospital for an extended period and/or has had multiple medication changes sometimes made by multiple medical teams. This, in part, may explain why there is often incomplete documentation of medication changes [10]. Ward-based pharmacists in Australia and other countries routinely perform medication reconciliation on discharge, which includes identifying medication changes and communicating them to the patient [10]. Yet this pharmacist-generated information is not routinely shared with primary care physicians. A study by Ooi et al. [24] reported that the option to receive a pharmacist-prepared summary of medication changes made in the hospital was an approach that many primary care physicians preferred and were satisfied with. In our study, when pharmacists contributed to discharge summaries, around 80% of clinically significant medication changes were stated in the EDS, and around two-thirds were both stated and explained. This is a major improvement compared to our baseline data, and other studies evaluating discharge summaries prepared without pharmacist involvement, where around 50% or fewer in-hospital medication changes are typically noted or explained [8,9,10,16,27].

Despite the clear benefits of this intervention, it is important to recognise the additional workload and time implications for pharmacists. This highlights the importance of an adequate hospital pharmacist workforce and resources to sustain it. Pharmacists reported that verifying and contributing to the EDS required, on average, 5 min of their time, and up to 16 min for more complex discharges. When patient turnover was high, reviewing and verifying discharge summaries on top of existing duties, without any additional staffing resource, was difficult for pharmacists in our study, as evidenced by the fact that fewer than 50% of EDSs were verified in the 2015 post-intervention audit. Although the intervention only required a few minutes per patient, when a pharmacist is managing up to 10 discharges per day this is a significant additional workload. In the aged care wards, where length of stay was longer, and hence the number of discharges per day was lower, around two-thirds of EDSs were verified and the pharmacists were able to sustain that level of contribution when it was incorporated into usual care. The intervention was not able to be sustained beyond the pilot intervention period in wards where turnover was high. 

Staffing and time constraints have been reported as challenges to the preparation of accurate and clear medication discharge information [28]. A recent study by Wilcock et al. [20] demonstrated sustained benefits of pharmacists improving the quality of discharge summaries and information on medication changes in their three-year audit but noted that pharmacists only contributed to 25% of discharge summaries. The expansion of pharmacists’ roles in the discharge process will require additional workflow and workload considerations [29,30]. There is evidence that pharmacist medication reconciliation interventions at the time of discharge from hospital can be cost saving, through the avoidance of adverse medication events and re-presentations to hospital [31], which suggests that health services should invest in adequate pharmacist resourcing to enable this to be consistently delivered. Expansion of clinical pharmacy services to include weekends would also enable more consistent delivery of the intervention—at the study hospital, there was a reduced clinical pharmacy service on weekends, which meant that contributing to discharge summaries was usually not feasible for patients discharged on those days. There is evidence that ward-based pharmacy technicians can free up pharmacists’ time for clinical care [32], and better utilisation of technicians may be another approach towards improving discharge summaries that could be explored in future studies.

Aside from workload and pharmacist resourcing, other barriers that sometimes prevented pharmacists from contributing to the EDS related to logistics and timing, and the need to coordinate with the medical team to ensure the EDS was not locked or signed off too soon. Greater integration of pharmacists into medical teams could help to overcome this barrier. Models involving the addition of clinical pharmacists into patient care teams have improved the completeness and accuracy of discharge information and decreased medication errors [18,23,33]. However, it should be acknowledged that this requires a close working relationship and shared understanding regarding the roles and responsibilities of members of the healthcare team [34].

It has been shown that active participation of pharmacists in multidisciplinary clinical teams is highly appreciated by physicians and saves medical staff time [25,35]. While the intervention described in our study required additional pharmacist time, anecdotal feedback from hospital physicians suggested that it saved them time, particularly time spent working out what medication changes had occurred, when they occurred, and why.

A recent commentary from two Australian physicians questioned the exclusive use of pharmacists to prepare medication information in medical discharge summaries [36], citing concerns about isolating medication reconciliation from other aspects of the medical discharge plan and the fact that not all discharges occur within pharmacist working hours. However, they went on to highlight the importance of involving pharmacists in medical teams to support physicians to ensure accurate information in discharge summaries, which is consistent with our model in which pharmacists and physicians collaborated to prepare EDS medication information.

This study has some limitations. EDSs were evaluated using retrospective audits. We may have under-estimated the proportion of EDSs that pharmacists reviewed, because pharmacists were instructed not to create an addendum when an EDS was signed off by a physician before the pharmacist reviewed it if there were no discrepancies or the discrepancies were low risk. In such cases, we were unable to ascertain whether the pharmacist reviewed the EDS. Decisions to create EDS addenda were left to the pharmacists’ professional judgement, so there may have been variation between pharmacists regarding how often they created addenda. It is also possible that reluctance to create addenda may have reduced the effectiveness of the intervention, however this was not explored. The use of final year pharmacy students for the 2017 audit, versus pharmacists for the earlier audits, may have led to differences in data collection. To mitigate this risk, a standardised data collection form was used across all audits, the students were trained and supervised by study investigators, including working through several cases with an investigator, and random data-checks were undertaken. There were more medication changes in the 2017 aged care sample compared to the previous samples (median seven versus five changes per patient). We do not believe this would have affected the conclusions of our study, because if the higher number of medication changes had any effect on our findings it would most likely have biased the study toward the negative (i.e., made it harder to demonstrate a benefit from the intervention), because more medication changes creates increased opportunity for discharge summary omissions and other discrepancies.

## 5. Conclusions

A pharmacist–physician collaborative model for preparing and verifying medication information in EDSs led to significantly fewer medication list discrepancies and a significant improvement in the communication of medication changes. As this model was added on to pharmacists’ existing clinical workloads, the pharmacists were not able to contribute to, or verify, every EDS, and the intervention was not able to be sustained on all wards. Additional resources would be required, ideally combined with increased pharmacist integration into medical teams, to enable consistent delivery of the intervention.

## Figures and Tables

**Table 1:** Reasons for exclusion.

	(a) All Pilot Intervention Wards		(b) Aged Care Wards only		
	Baseline (2014)	Post-Intervention (2015)	Baseline (2014)	Post-Intervention (2015)	Post-Intervention (2017)
No completed electronic discharge summaries (EDSs) in Cerner	8	11	4	1	4
Scanned copy of pharmacist-reviewed and reconciled discharge prescription missing, incomplete ^ or illegible *	14	10	2	4	7
Pharmacist-verified ‘Medication History on Admission’ form absent from medical record	6	2	1	1	1
Patient discharged to another hospital	0	0	0	0	9
Total	22	24	5	7	20

^ Discharge prescriptions often spread over multiple pages. Sometimes one or more pages were inadvertently missed when the prescriptions were manually scanned by the pharmacy department. * Security features on Australian Government Pharmaceutical Benefits Scheme prescription paper (shading/watermark) resulted in scanned images of discharge prescriptions sometimes becoming illegible. Note: Some patients met more than one exclusion criteria.

**Table 2:** Patient demographics (all pilot intervention wards).

Demographics	Baseline (2014) (n = 93)	Post-Intervention (2015) (n = 96)
Age (years), median (IQR)	79 (65–85)	81 (70–86)
Gender		
Male, number (%) Female, number (%)	40 (43) 53 (57)	47 (49) 49 (51)
Length of admission (days), median (IQR)	6 (4–21)	6 (3–17)
Number of regular medications on discharge, median (IQR)	9 (5–12)	8 (5–11)
Number of changes to pre-admission medication regimen made in hospital, median (IQR)	4 (3–7)	4 (2–6)
Number of clinically significant changes to pre-admission medication regimen made in hospital, median (IQR)	4 (2–6)	3 (2–5)

IQR = interquartile range.

**Table 3:** Accuracy of discharge summaries pre- and post-intervention (all pilot intervention wards).

	Baseline (2014) (n = 93)	Post-Intervention (2015) (n = 96)	*p*-Value
Total number of EDS medication list discrepancies	129	53	N/A
Proportion of EDSs with one or more medication list discrepancies, n (%)	62/93 (67)	36/96 (38)	<0.001
Median (IQR) number of EDS medication list discrepancies per patient	1 (0–2)	0 (0–1)	<0.001
Total number of clinically significant medication list discrepancies	63	15	N/A
Proportion of EDSs with one or more clinically significant medication list discrepancies, n (%)	40/93 (43)	14/92 (15)	<0.001
Proportion of clinically significant medication changes that were stated in the EDS, n (%)	222/417 (53)	296/366 (81)	<0.001
Proportion of clinically significant medication changes that were stated AND explained in the EDS, n (%)	206/417 (49)	245/366 (67)	<0.001
Proportion of EDSs with evidence of pharmacist verification, n (%)	N/A	45/96 (47)	N/A

EDS = electronic discharge summary; N/A = not applicable; IQR = interquartile range.

**Table 4:** Patient demographics (aged care wards).

	Baseline (2014) (n = 41)	Post-Intervention (2015) (n = 42)	Post-Intervention (2017) (n = 76)
Age (years), median (IQR)	84 (80–90)	82 (76–84)	82 (72–87)
Gender			
Male, number (%) Female, number (%)	17 (41) 24 (59)	24 (57) 18 (43)	31 (41) 45 (59)
Length of admission (days), median (IQR)	20 (7–29)	17 (7–36)	33 (19–51)
Number of regular discharge medications, median (IQR)	9 (7–12)	9 (6–11)	9 (5–13)
Number of medication changes, median (IQR)	5 (3–8)	5 (3.25–6)	7 (6–11.25)
Number of clinically significant medication changes, median (IQR)	5 (3–7)	5 (3–6)	7 (5–11)

IQR = interquartile range.

**Table 5:** Accuracy of discharge summaries pre- and post-intervention with two-year follow-up (aged care wards).

	Baseline (2014) (n = 41)	Post-Intervention (2015) (n = 42)	Post-Intervention (2017) (n = 76)	*p*-Value (All Groups)
Total number of EDS medication list discrepancies	43	15	58	N/A
Proportion of EDSs with one or more medication list discrepancies, n (%)	26/41 (63)	11/42 (26) *	27/76 (36) *	0.001
Median (IQR) number of EDS medication list discrepancies per patient	1 (0–2)	0 (0–1) *	0 (0–1) *	<0.001
Total number of clinically significant medication list discrepancies	23	6	27	N/A
Proportion of EDSs with one or more clinically significant medication list discrepancies, n (%)	18/41 (44)	5/42 (12) *	18/76 (24) *	0.003
Proportion of clinically significant medication changes that were stated in the EDS, n (%)	109/219 (50)	185/212 (87) *	464/612 (76) *^,^#	<0.001
Proportion of clinically significant medication changes that were stated AND explained in the EDS, n (%)	94/219 (43)	141/212 (67) *	403/612 (66) *	<0.001
Proportion of EDSs with evidence of pharmacist verification, n (%)	N/A	27/42 (64)	52/76 (68)	0.65

N/A = not applicable; IQR = interquartile range; * *p* < 0.05 vs. 2014; # *p* < 0.05 vs. 2015.
